# Peculiarity of Two Thermodynamically-Stable Morphologies and Their Impact on the Efficiency of Small Molecule Bulk Heterojunction Solar Cells

**DOI:** 10.1038/srep13407

**Published:** 2015-08-28

**Authors:** Nuradhika Herath, Sanjib Das, Jong K. Keum, Jiahua Zhu, Rajeev Kumar, Ilia N. Ivanov, Bobby G. Sumpter, James F. Browning, Kai Xiao, Gong Gu, Pooran Joshi, Sean Smith, Valeria Lauter

**Affiliations:** 1 Quantum Condensed Matter Division, Oak Ridge National Laboratory, Oak Ridge, TN 37831, USA; 2 Department of Electrical Engineering and Computer Science, University of Tennessee, Knoxville, TN 37996, USA; 3 Center for Nanophase Materials Sciences, Oak Ridge National Laboratory, Oak Ridge, TN 37831, USA; 4 Chemical and Engineering Materials Division, Oak Ridge National Laboratory, Oak Ridge, TN 37831, USA; 5 Computer Science and Mathematics Division, Oak Ridge National Laboratory, Oak Ridge, TN, 37831, USA; 6 Materials Science and Technology Division, Oak Ridge National Laboratory, Oak Ridge, TN 37831, USA; 7 School of Chemical Engineering, UNSW Australia, Sydney, NSW 2052, Australia

## Abstract

Structural characteristics of the active layers in organic photovoltaic (OPV) devices play a critical role in charge generation, separation and transport. Here we report on morphology and structural control of *p*-DTS(FBTTh_2_)_2_:PC_71_BM films by means of thermal annealing and 1,8-diiodooctane (DIO) solvent additive processing, and correlate it to the device performance. By combining surface imaging with nanoscale *depth-sensitive* neutron reflectometry (NR) and X-ray diffraction, three-dimensional morphologies of the films are reconstituted with information extending length scales from nanometers to microns. DIO promotes the formation of a well-mixed donor-acceptor vertical phase morphology with a large population of *small p*-DTS(FBTTh_2_)_2_ nanocrystals arranged in an elongated domain network of the film, thereby enhancing the device performance. In contrast, films without DIO exhibit three-sublayer vertical phase morphology with phase separation in agglomerated domains. Our findings are supported by thermodynamic description based on the Flory-Huggins theory with quantitative evaluation of pairwise interaction parameters that explain the morphological changes resulting from thermal and solvent treatments. Our study reveals that vertical phase morphology of small-molecule based OPVs is significantly different from polymer-based systems. The significant enhancement of morphology and information obtained from theoretical modeling may aid in developing an optimized morphology to enhance device performance for OPVs.

Organic photovoltaics (OPVs) are promising light conversion technologies expecting to meet the demands of low manufacturing costs, green technology, and efficient operation under low-light conditions^1^,^2^. Major breakthroughs in OPV performance have been achieved by incorporating new conjugated polymers or small molecules into bulk heterojunction (BHJ) devices enabling further optimization of active layer morphology^3^,^4^,^5^,^6^,^7^. During solution casting of the OPV layer, electron donor (ED) and acceptor (EA) molecules undergo self-assembly leading to a bicontinuous, interpenetrating network of ED and EA phases, which provide pathways for the transport of electrons and holes. The BHJ ED-EA network with a length scale ranging from nano- to mesoscale is crucial to the power conversion efficiency (PCE) of OPVs^8^. Several successful approaches of optimizing the network morphology include thermal or solvent annealing^9^,^10^,^11^, casting from a high boiling point or from a binary solvent, or by introducing processing additives^12^,^13^,^14^,^15^, all of which improve exciton diffusion-dissociation and charge transport, leading to higher PCE^16^,^17^,^18^,^19^,^20^.

Recently, low band gap conjugated polymer/fullerene^21^,^22^ and small molecule/fullerene^23^,^24^,^25^,^26^,^27^,^28^,^29^,^30^,^31^ blends have shown more promising results. The latter system is known to be easier to purify, and the devices show consistently better batch-to-batch reproducibility, higher crystallinity and better performance^28^,^32^,^33^. Devices based on the ED:EA system of 7,7′-[4,4-Bis (2-ethylhexyl)-4*H*-silolo[3,2-*b*:4,5-*b*′]dithiophene-2,6-diyl]bis[6-fluoro-4-(5′-hexyl-[2,2′-bithiophen]-5-yl)benzo[*c*][1,2,5]thiadiazole]) (*p-*DTS(FBTTh_2_)_2_ and [6,6]-Phenyl C_72_ butyric acid methyl ester (PC_71_BM) have demonstrated PCEs as high as ~9%^25^. Thermal annealing of *p-*DTS(FBTTh_2_)_2_:PC_71_BM further improves the performance through the formation of wire-like 2D crystal domains of *p-*DTS(FBTTh_2_)_2_ reaching ~100 nm in length^13^. The results obtained by single crystal x-ray diffraction (SCXRD) and grazing incidence wide angle x-ray scattering (GIWAXS) indicated that *p-*DTS(FBTTh_2_)_2_ molecules grew into two-dimensional (2D) columnar arrays with increased π–π overlapping, which leads to improved intermolecular charge transport, and enhanced value of PCE^13^. A processing additive, 1,8-diiodooctane (DIO) enables formation of a well interconnected network of crystalline *p-*DTS(FBTTh_2_)_2_ domains with of ~30 nm^14^. It was also shown that the addition of 0.4 vol% of DIO results in different coherent lengths of PC_71_BM and *p-*DTS(FBTTh_2_)_2_ domains^34^. Recent advances in structural study of the polymer/fullerene system have identified the presence of mixed phases containing fullerene molecules dispersed in the donor phase^35^,^36^. While pure donor and acceptor phases reduce charge recombination by pushing holes away from electrons and, thus, enhancing device performances, the role of a third phase remains unclear^35^,^36^. Formation of the mixed phases may be expected in SM OPV as well as *p*-DTS(FBTTh_2_)_2_ and PC_71_BM phase-separation during solidification. If one blending component prefers the air/film, bulk or film/substrate interface it may lead to complex vertical phase stratification, including pure or mixed phases with a morphology different from bulk phase of BHJ. Thus, not only the in-plane structure, but also the vertical structure of the SM OPV should be investigated in detail. Highly penetrative and nondestructive neutron reflectometry is an ideal tool for profiling the buried phases and interfacial morphology of *p*-DTS(FBTTh_2_)_2_:PC_71_BM blended films in the direction perpendicular to the film surface down to the substrate. The high contrast in neutron scattering length density (*n*SLD) between *p*-DTS(FBTTh_2_)_2_ (1.33 × 10^−6^ Å^−2^) (see experimental section) and PC_71_BM (4.3 × 10^−6^ Å^−2^)^37^ allows clear distinction of the components. While the depth phase morphologies of various conjugated polymer: fullerene derivative OPVs have been successfully described using neutron reflectometry^38^,^39^,^40^,^41^,^42^,^43^ or by depth profiling secondary ion mass spectroscopy (DSIMS)^44^, investigation of the depth phase morphology of SM OPV has not been reported. Here, we use neutron reflectometry^43^ to unfold the depth phase morphology of *p*-DTS(FBTTh_2_)_2_ and PC_71_BM blend and complemented with the use of absorption and photoluminescence spectroscopy, atomic force microscopy (AFM) and X-ray diffraction (XRD) characterizations to correlate the morphology to the OPV device performance after thermal annealing and DIO additive processing. Our results reveal that depth profiles of SM-based systems are different from the polymer-based systems. A thermodynamic description based on Flory-Huggins theory was used to better understand the effect of DIO and thermal annealing on the structure and morphologies of thin active layers, and its relationship with the device performances. Our combined experimental and theoretical approach allows us to demonstrate correlations between the morphology and device performance, as well as to explain origin of different morphologies.

## Results and Discussion

**Figure 1:**
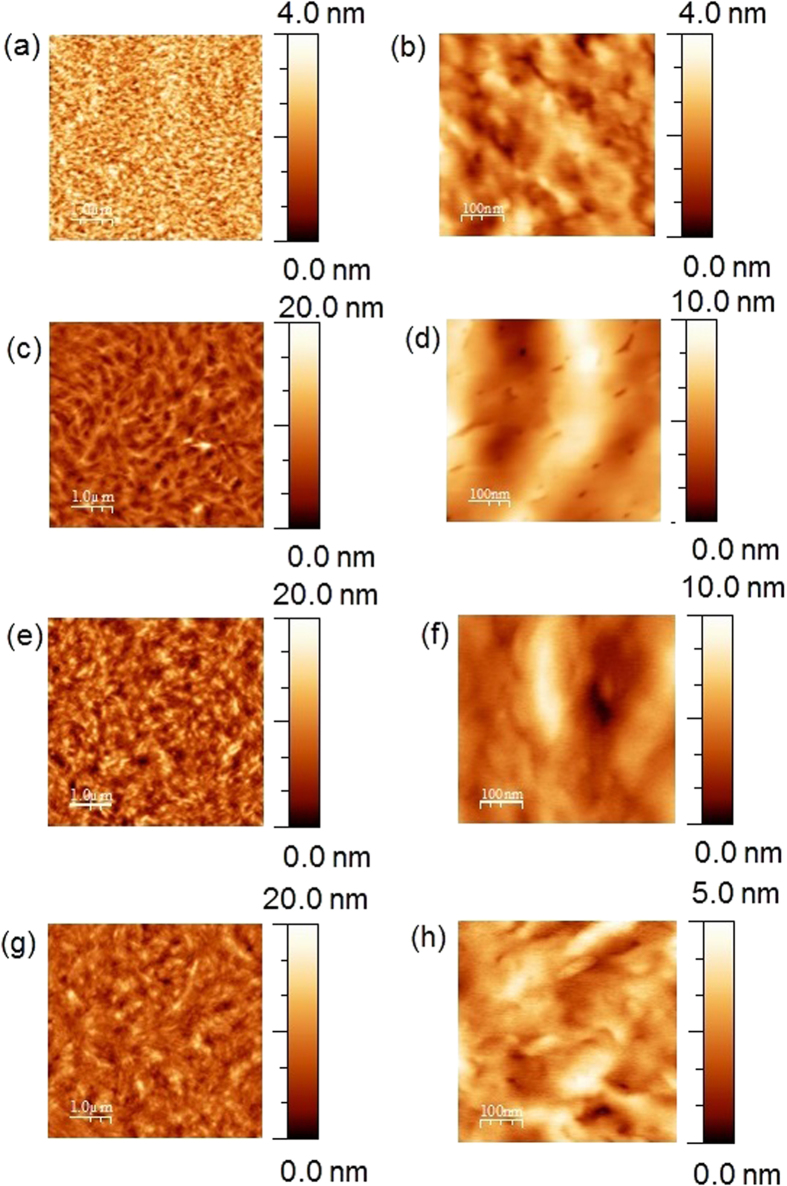
(Left) Atomic force microscopy (AFM) images of the *p*-DTS(FBTTh_2_)_2_:PC_71_BM films (**a**) as prepared (**c**)thermally annealed at 100 °C. (**e**) with 0.25 vol% DIO (**g**) with 0.25 vol% DIO thermally annealed at 80 °C (Right) High resolution images of *p*-DTS(FBTTh_2_)_2_:PC_71_BM films.

Thin films were fabricated by spin casting a solution of *p-*DTS(FBTTh_2_)_2_:PC_71_BM (1.5:1 wt.%)/chlorobenzene onto a sapphire (Al_2_O_3_) or quartz (SiO_2_) substrate. The surface morphologies of *p-*DTS(FBTTh_2_)_2_:PC_71_BM thin films were investigated with AFM in tapping mode. Figure 1 shows AFM images of (a) as-cast, and (c) thermally annealed *p-*DTS(FBTTh_2_)_2_:PC_71_BM films and films with (e) 0.25 vol% DIO and (g) after annealing at 80 °C. The surface of the as-cast sample, as evident from Fig. 1(a), consists of *p-*DTS(FBTTh_2_)_2_ and PC_71_BM molecules in a random arrangement as a uniform mixture. The root mean square roughness (*r*_*RMS*_) of the sample increases from 0.59 nm to 1.75 nm after thermal treatment. The surface of the annealed film consists of domains, which are approximately 500 nm to 1 μm long. The morphology of the surface of the sample with 0.25 vol% DIO (Fig. 1(e)) is drastically different from (a) and (b) without DIO. It is composed of long wire-shape domains extending up to ~200 nm. After the thermal annealing (Fig. 1(g)), the *r*_*RMS*_ of the films decreases from ~2.39 nm to ~2.27 nm.

**Figure 2:**
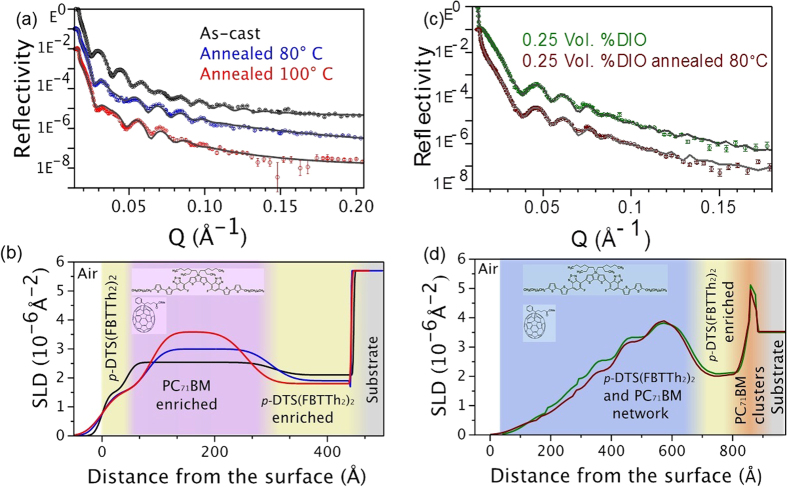
(**a**) Neutron reflectivity data of Al_2_O_3_\\*p-*DTS(FBTTh_2_)_2_:PC_71_BM BHJ test structures as prepared and annealed at 80 °C, 100 °C. Experimental data are shown as symbols and the fits as lines. (**b**) Scattering length density (SLD) profiles as a function of distance from the surface obtained after the fits to the experimental NR data of Fig 1(a). (**c**) Neutron reflectivity data of SiO_2_\\*p-*DTS(FBTTh_2_)_2_:PC_71_BM BHJ with 0.25 V% DIO device as prepared and annealed at 80 °C. Experimental data are shown as symbols and the fits as lines. (**d**) Scattering length density (SLD) profiles as a function of distance from the surface from fitting the experimental NR data of (**c**).

Figure 2 shows the vertical phase morphology of *p-*DTS(FBTTh_2_)_2_:PC_71_BM films, which was investigated using neutron reflectometry. In devices, the active layer is usually spun cast on amorphous PEDOT:PSS layer. However, in previously reported neutron reflectivity experiments, the studies have been performed using BHJ films grown directly on the substrate^38^,^40^,^43^,^45^, which have similar surface properties as Al_2_O_3_ or SiO_2_ substrates used in these experiments. Therefore, we primarily compared the neutron reflectometry results from samples directly spin cast on SiO_2_ and Al_2_O_3_ substrates (Fig. S1) and that on PEDOT:PSS layer (Fig. S2). The active layer vertical phase morphologies of the samples are similar indicating that BHJ films fabricated directly on substrates would accurately reflect the vertical phase morphology of the OPV devices. Figure 2(a) presents neutron reflectivity (NR) for as-cast, and thermally annealed films at 80 °C and 100 °C as a function of out-of-plane momentum transfer, *Q*_*z*_ of specular reflections. Here, *Q*_*z*_ = (4*π*/*λ*) sin*α*_*i*_ with *λ* and *α*_*i*_ being the wavelength and the incident angle of neutron beam, respectively. The associated fit of the data obtained based on Parratt recursion formalism is shown as solid line^46^. We estimated the value of neutron scattering length density (*n*SLD) for *p-*DTS(FBTTh_2_)_2_ to be 1.33 × 10^−6^ Å^−2^ (see experimental methods). The *n*SLD value of PC_71_BM was found to be 4.34 × 10^−6^ Å^−2^ based on the bulk density of 1.5 g/cm^3^ of PCBM and neutron scattering lengths of the constituent elements^37^. Figure 2(b) shows the *n*SLD profiles of the best fit to the reflectivity data. A detailed description of models used to fit the data is given in the Supplementary Materials (Fig. S3). From the fits, the total thickness of the as-cast film was found to be ~45 nm. The *n*SLD profiles in Fig. 2(b) reveal three distinct layers with the composition characterized by different *n*SLDs: 1) top surface layer in contact with the air/film interface, 2) a bottom layer interfacing with the substrate, and 3) a bulk layer sandwiched by the two interfacial layers. It also shows that the *n*SLD of the sandwiched layer increases with annealing temperature while that of the bottom layer decreases. The changes in *n*SLDs of the bulk and bottom layer are associated with the inter-diffusion of *p-*DTS(FBTTh_2_)_2_ and PC_71_BM. Remarkably, the *n*SLD of top interfacial layer remained almost unchanged regardless of thermal annealing. These results indicate that *p-*DTS(FBTTh_2_)_2_ prefers the two interfacial regions, whereas, PC_71_BM tends to diffuse to the bulk layer. To complement the depth phase morphology deduced by NR data fits, we performed X-ray reflectivity measurements shown in Fig. S4. Although the contrast between X-ray SLDs (*x*SLDs) of *p*-DTS(FBTTh_2_)_2_ and PC_71_BM is relatively low as compared to *n*SLDs, we observed an obvious change in *x*SLD with thermal annealing. The total number of sub-layers and the thermally induced changes such as enhancement of *x*SLD in the bulk layer and reduction in the film thickness are consistent with the NR results.

Figure 2(c) shows the neutron reflectivity profiles of as-cast and annealed *p-*DTS(FBTTh_2_)_2_:PC_71_BM films prepared using 0.25 vol.% DIO. From the NR fits, the *n*SLD profiles were extracted and are depicted in Fig. 2(d). The *n*SLD profile of the as-cast film with 0.25 vol.% DIO is remarkably different from the one without additive. Although it shows the presence of *p-*DTS(FBTTh_2_)_2_ enriched bottom interfacial layer, its width is about 3 to 4 times thinner than in the samples without DIO. Further, a thermal annealing the film with DIO at 80 °C induces only small change in the *n*SLD profile. This is different from the change in *n*SLD profile for as-cast *p-*DTS(FBTTh_2_)_2_:PC_71_BM film and sample annealed at 80 °C (Fig. 2(b)). It indicates that no significant inter-diffusion of *p-*DTS(FBTTh_2_)_2_ and PC_71_BM occurs by the thermal annealing. This observation is consistent with our AFM data where the films show densely packed structure with reduced surface roughness. Furthermore, at the bottom substrate/film interfaces, NR revealed a ~20 nm thick layer with a high *n*SLD value of ~6 × 10^−6^ Å^−2^ in both as-cast and annealed samples with DIO. The peaks correspond to high-density PC_71_BM clusters formed in the samples with DIO^47^. Upon thermal annealing, the *n*SLD of the profile increased resulting densely packed films.

These results show that presence of DIO results in much more evolved film morphology, which is close to the equilibrium state than that of the film spun-cast without DIO. Hence, thermal annealing only results in small refinement in the structure with negligible difference from as spun-cast film^45^. It is evident from the *n*SLD profiles that, for as-cast samples, accumulation of the ED material at the air/film interface was enhanced with thermal annealing, which was previously observed using X-ray photon spectroscopy (XPS) for *p*-DTS(FBTTh_2_)_2_ material^11^. By contrast, polymer: fullerene blend systems exhibit accumulation of PCBM (PC_61_BM or PC_71_BM) at film/substrate and air/film interfaces which is believed to be responsible for enhanced electron extraction^38^,^39^. Hence, the layer morphology of *p-*DTS(FBTTh_2_)_2_:PC_71_BM appears very differently from polymer: fullerene blend systems.

**Figure 3:**
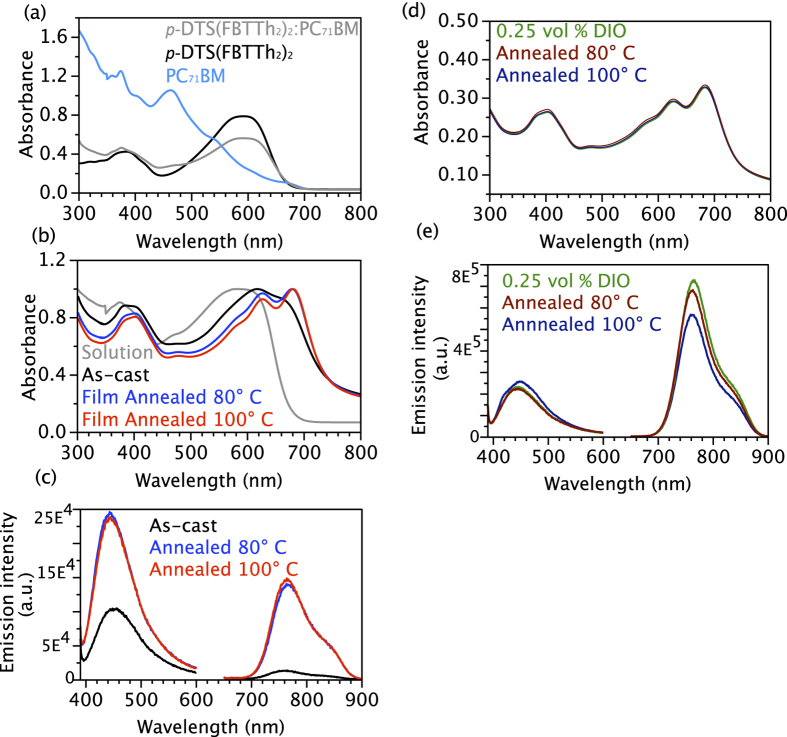
(**a**) UV-Visible absorption spectra of *p*-DTS(FBTTh_2_)_2_, PC_71_BM and blend of *p*-DTS(FBTTh_2_)_2_:PC_71_BM in chlorobenzene solvent (**b**) Absorption spectra of *p*-DTS(FBTTh_2_)_2_:PC_71_BM blend solution and cast films under thermal annealing. The absorption intensities are normalized. (**c**) Photoluminescence spectra of as-cast *p*-DTS(FBTTh_2_)_2_:PC_71_BM film after thermal annealing at 80 °C and 100 °C. (**d**) UV- Visible absorption and (**e**) photoluminescence spectra of *p*-DTS(FBTTh_2_)_2_:PC_71_BM with 0.25 vol.% DIO after thermal annealing at 80 °C.

The structural evolution of SM in solutions and films can be correlated with the changes in electronic and photoluminescence spectra. We assigned the broad absorption peaks at 390 and 600 nm in chlorobenzene (CB) solutions of *p*-DTS(FBTTh_2_)_2_, PC_71_BM to π–π* transition of *p-*DTS(FBTTh_2_)_2_, while 370 nm and 460 nm peaks were ascribed to the PC_71_BM, Fig. 3(a). Upon spin casting of the *p-*DTS(FBTTh_2_)_2_:PC_71_BM solution into film, a bathochromic shift of all absorption peaks is observed. As-cast thin film exhibits 550, 615 and 680 nm broad vibronic peaks of π–π stacked *p-*DTS(FBTTh_2_)_2_ aggregates, assigned to A_0→2_, A_0→1_ and A_0→0_ transitions respectively^48^. The ratio of A_0→1_/A_0→0_ > 1 is indicative of dominating inter-chain coupling in π–π stacked *p-*DTS(FBTTh_2_)_2_ aggregates^48^. Upon thermal annealing at 80 °C and 100 °C, the A_0→2_, A_0→1_ and A_0→0_, vibronic peaks become more pronounced and show small bathochromic shift, indicative of an enhanced crystalline ordering in SM aggregates. The reduced intensity ratio of A_0→1_/A_0→0_ < 1 is usually attributed to planarization of *p-*DTS(FBTTh_2_)_2_ molecules, with intra-chain interactions dominating over inter-chain coupling in SM aggregates. The PL spectra of the DTS(FBTTh_2_)_2_:PC_71_BM film show two broad emission bands with maxima at 460 nm (2.7 eV) and 765 nm (1.6 eV), when excited at 320 nm and 580 nm, respectively. The 1.6 eV emission correlates with the transition between LUMO and HOMO of *p-*DTS(FBTTh_2_)_2_ aggregates, while the 2.7 eV band was assigned to the transition from LUMO_*p-*DTS(FBTTh2)2_ to HOMO_PC71BM_^25^. Annealing leads to increased PL intensity of 1.6 eV and 2.7 eV peaks, which correlates well with the changes in absorption spectra, and is probably related to the increase in *p-*DTS(FBTTh_2_)_2_ aggregate π−π stacking and the structure of *p-*DTS(FBTTh_2_)-PC_71_BM interface respectively.

The absorption spectra of as-cast and annealed *p-*DTS(FBTTh_2_)_2_:PC_71_BM films are shown in Fig. 3(d). In addition to a broad ~400 nm peak, a progression of vibronic peaks was detected at 580 nm, 625 nm, and 680 nm, which are assigned to A_0→2_, A_0→1_, and A_0→0_ transitions, respectively. The ratio of A_0→1_ to A_0→0_ peak intensities was found to be less than one which does not change upon annealing, indicating that high order of π−π stacking and molecular planarization in SM aggregate in the presence of DIO are achieved already at room temperature. PL spectra show same peaks without DIO. However, while the intensity of broad 2.7 eV peak did not change, the intensity of low energy emission peak at 1.6 eV increased by a factor of five compared to annealed *p-*DTS(FBTTh_2_)_2_:PC_71_BM films without DIO. The improved PL intensity indicates that defect-related non-radiative transition is suppressed in the case of DIO containing films, due to better ordering of SM aggregates. Both electronic absorption and PL spectra of annealed DIO containing *p-*DTS(FBTTh_2_)_2_:PC_71_BM films show that thermodynamically metastable molecular ordering was achieved already at room temperature. This metastable molecular ordering is consistent with the unchanged vertical phase morphology of DIO containing *p-*DTS(FBTTh_2_)_2_:PC_71_BM films observed in NR experiments.

**Figure 4:**
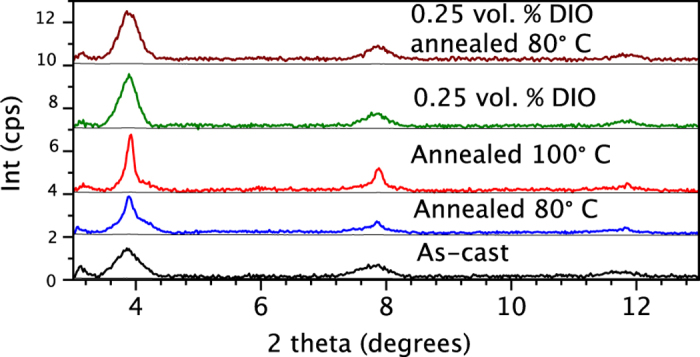
Out of plane X-ray diffraction scans of *p*-DTS(FBTTh_2_)_2_:PC_71_BM blends. 2θ = 3.98°, 7.88° and 11.90°. With thermal annealing, the intensity of the peaks is enhanced. Addition of 0.25 vol% DIO generated broader peak with higher intensities.

To gain insights into the effect of DIO and thermal annealing on the molecular packing and crystallinity of *p-*DTS(FBTTh_2_)_2_:PC_71_BM films, we conducted X-ray diffraction (XRD) measurements. Figure 4 shows XRD patterns for as-cast and annealed films with and without DIO. All films exhibit a peak at *2θ* = ~3.90°, which is associated with the (001) reflection of *p-*DTS(FBTTh_2_)_2_ crystal. Higher order (002) and (003) reflections observed at ~7.88°, and ~11.74° manifests that a fraction of *p-*DTS(FBTTh_2_)_2_ chains are highly-ordered aggregates within films^14^. Upon annealing of thin films, the XRD peak intensity and sharpness increase, indicating an increase in ordering of *p-*DTS(FBTTh_2_)_2_ aggregates. Using the Scherrer equation, the size of crystalline domain of SM aggregate was calculated along with the peak areas for (001) reflection of *p-*DTS(FBTTh_2_)_2_ crystal (Table 1). The crystalline domain size in as-cast films were found to be ~22.8 nm, which increases to 41.2 nm and 134.7 nm after thermal annealing at 80 °C and 100 °C, respectively. The integrated (001) peak area of the sample annealed at 100 °C is greater than that of the as-cast sample, suggesting an increased population of crystalline domains of *p-*DTS(FBTTh_2_)_2_ in the annealed *p-*DTS(FBTTh_2_)_2_:PC_71_BM film (Table 1). Remarkably the integrated area of (001) peak of as-cast *p-*DTS(FBTTh_2_)_2_:PC_71_BM films with DIO is about three times larger than that without DIO. Interestingly, the crystalline domain size of sample is ~24.6 nm, which is a ~2 nm increase to that of film without DIO. We conclude that DIO drastically increases the population of *p-*DTS(FBTTh_2_)_2_ crystal domains already in as-cast films, and only slightly increases the crystal domain size. Thermal annealing of DIO-containing *p-*DTS(FBTTh_2_)_2_:PC_71_BM films increases the size and the population of *p-*DTS(FBTTh_2_)_2_ crystalline domains. We also observe that the size of crystalline domains in annealed (80 °C) DIO-containing *p-*DTS(FBTTh_2_)_2_:PC_71_BM films is smaller by a factor of 1.6 compared to that in annealed *p-*DTS(FBTTh_2_)_2_:PC_71_BM films without DIO while the population of crystals increases by a factor of 3. Larger crystal domain size in annealed *p-*DTS(FBTTh_2_)_2_:PC_71_BM films suggests that excitons have to travel longer distances to reach D/A interface, which is detrimental for achieving high PCEs.

**Figure 5:**
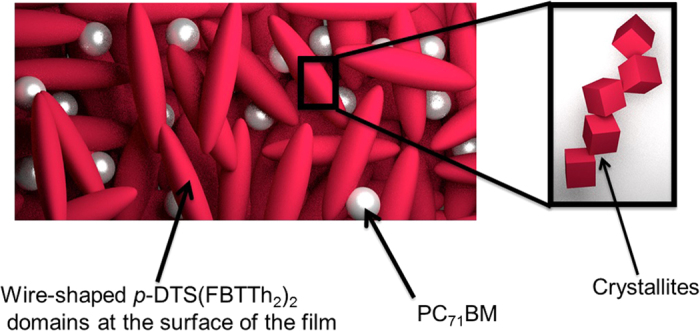
Schematic illustration of the change of surface morphology with addition of DIO. With 0.25 vol% DIO, *p*-DTS(FTTh_2_)_2_ shows to generate ~200 nm long wire-shaped domains structures, which are made up of ~26 nm crystallites. This illustration is drafted using data obtained from AFM, neutron reflectometry and XRD measurements.

Combining results of AFM, XRD and neutron reflectometry allows visualization of the surface and depth morphology of *p-*DTS(FBTTh_2_)_2_:PC_71_BM thin films, which is shown in Fig. 5. The drawing shows changes in the blend microstructure upon thermal annealing and addition of DIO. Formation of wire-shaped domains in the films generated uneven surfaces, with the roughness of ~20 nm. Our NR data reveals that surface of films consist of *p-*DTS(FBTTh_2_)_2_ and PC_71_BM. These wire-shaped features can be attributed to *p-*DTS(FBTTh_2_)_2_ domians^13^. In addition, the lengths of the wires are much longer than the crystallite sizes obtained from our XRD. This could be due to the fact that these wires are made of few crystallites connected together through π−π stacking.

**Figure 6:**
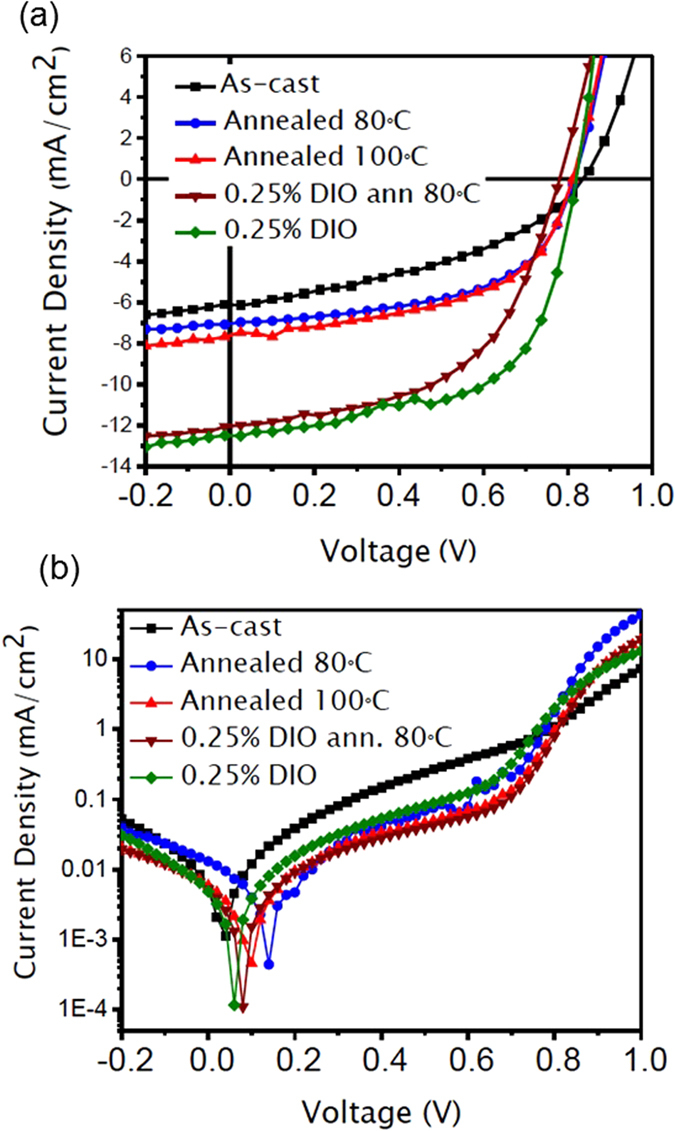
J-V characteristics of the *p-*DTS(FBTTh_2_)_2_:PC_71_BM BHJ solar cells at different processing conditions (**a**) under AM 1.5 G irradiation at 100 mW/cm^2^ (**b**) in the dark.

Figure 6 shows the current density versus voltage (*J-V*) curves of *p-*DTS(FBTTh_2_)_2_:PC_71_BM solar cells under AM 1.5 G, 100 mW/cm^2^ irradiation from a solar simulator. The photovoltaic parameters such as short circuit current density (*J*_SC_), open circuit voltage (*V*_OC_), fill factor (FF) and power conversion efficiency (PCE) are listed in Table 2, based on the statistical average of ten devices. The device consisting of *p-*DTS(FBTTh_2_)_2_:PC_71_BM active layer shows relatively low value of PCE, about 2.04%. After thermal annealing, the PCE increases to 3.18% at 80 °C and 3.48% at 100 °C. DIO-containing DTS(FBTTh_2_)_2_:PC_71_BM devices show higher performance with PCE of 4.95% for as-cast active layer, which increases to 5.27% after annealing at 80 °C. The improved performance of DIO-containing device results for the fact that the additive induces crystallization of size of SM aggregates with better spectral response as indicated by increase in PL^49^.

**Figure 7:**
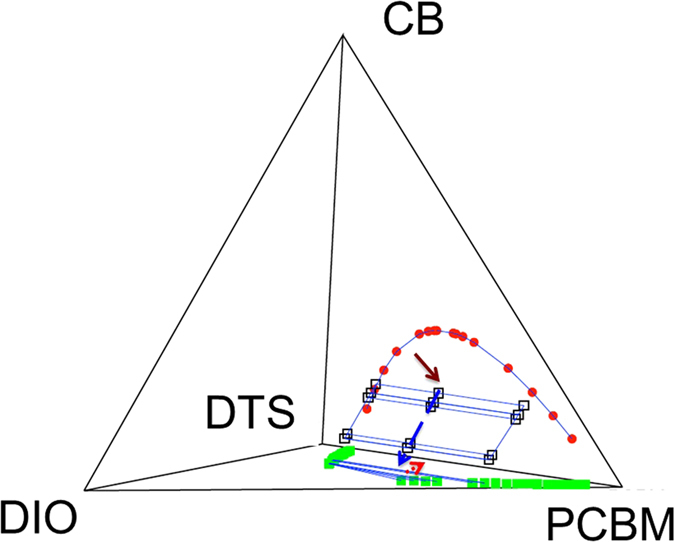
Phase diagram for the *p-*DTS(FBTTh_2_)_2_:PC_71_BM:CB:DIO system constructed using the Flory-Huggins theory showing different thermodynamic pathways in the presence and absence of DIO. In this figure, the label DTS represents *p-*DTS(FBTTh_2_)_2_ and the blue lines represent the tie lines.

To model the effect of annealing and of DIO additive on the phase separation in SM OPV system, we have constructed a phase diagram for the four-component system (*p-*DTS(FBTTh_2_)_2_:PCBM:CB:DIO) using Flory-Huggins theory,^50^ corroborated by large-scale molecular dynamics simulations^45^,^51^. Structural parameters for molecules used in the theory are shown in Table S1. Values of Flory’s χ_ij_ are summarized in Table 3, with the higher value indicating stronger repulsive interactions between *i* and *j* components. The quarternary phase diagram computed using the Flory-Huggins theory for the *p*-DTS(FBTTh_2_)_2_:PC_71_BM:CB:DIO system is shown in Fig. 7. The phase diagram highlights the different thermodynamic pathways to reach the same binary blend of *p*-DTS(FBTTh_2_)_2_ and PC_71_BM. One path is along the CB-PC_71_BM- *p*-DTS(FBTTh_2_)_2_ plane in Fig. 7, where movement towards the binary blend state point is driven by solvent (i.e., CB) evaporation. Treating *p*-DTS(FBTTh_2_)_2_ and PC_71_BM as oligomers with the degree of polymerization of 10 and 6, respectively, to account for disparity in molar volume with respect to the solvent CB (cf. Table S1) phase diagram can be readily constructed using the Flory-Huggins theory exhibiting a critical point at *ϕ*_*p-*DTS(FBTTh2)2_ = 0.44 and χ_DTS,PCBM_ = 0.26. The experimental system in this work contains 21 mg/ml of *p*-DTS(FBTTh_2_)_2_ mixed with 14 mg/ml of PC_71_BM, corresponding to a volume fraction of *p*-DTS(FBTTh_2_)_2_ equal to *ϕ*_*p-*DTS(FBTTh2)2_ = 0.60. An estimated value of χ_DTS,PCBM_ = 0.38 at room temperature using the group contribution method, segregation between *p*-DTS(FBTTh_2_)_2_ and PC_71_BM can be expected in the experimental *p*-DTS(FBTTh_2_)_2_:PC_71_BM mixture towards equilibrium binary phases. It agrees with the NR observation that *p*-DTS(FBTTh_2_)_2_ and PC_71_BM distribute heterogeneously along vertical direction of *p*-DTS(FBTTh_2_)_2_:PC_71_BM film annealed at different temperatures. For instance, the volume fractions of *p*-DTS(FBTTh_2_)_2_ in film annealed at 100 °C shows *ϕ*_*p-*DTS(FBTTh2)2_ = 0.8, 0.4 and 0.7 in surface, bulk and substrate layers, respectively. Given the simplistic nature of the Flory-Huggins theory, such a qualitative agreement with the experiments is quite remarkable.

Conversely, DIO-containing *p*-DTS(FBTTh_2_)_2_:PC_71_BM films show slight change in crystal domain size after thermal annealing, which is probably due to the change in miscibility of PC_71_BM in chlorobenzene after adding DIO. The miscibility of PC_71_BM is higher in the presence of DIO additives^15^. In this case, *p-*DTS(FBTTh_2_)_2_ might crystallize resulting in smaller but highly populated crystal growth. As the boiling point of DIO is much higher than CB, DIO could remain in a spun-cast film even after complete evaporation of CB. During spin coating, the DIO additive facilitates the formation of well-ordered structure, with donor and acceptor materials forming nano-scale domains, favorable for efficient charge transport. NR reveals the formation of a thin layer of PC_71_BM clusters at the film/substrate interface with addition of DIO. A 3-D tomography study conducted by Li *et al.* observed similar vertical elongated clusters for polymer, HXS-1 and PCBM based solar cells with 2.5 vol.% DIO^47^. According to their study, these clusters facilitate the charge transport and minimize the charge recombination of the devices. NR also reveals the formation of a *p-*DTS(FBTTh_2_)_2_ rich layer at the surface without DIO, without reduction of charge carrier mobility. This can be explained in terms of increased phase purity as evident by NR and XRD data, which facilitates the efficient charge transport through the devices observation and agrees well with previously published results^34^. Notably, the addition of DIO improves OPV performances, namely, changing value of FF from 38.84% to 58.15%,and the value of J_SC_ from 6.66 to 12.01, and leading to larger value of PCE = 5.27%.

Flory-Huggins theory also shed light on the effects of DIO additive with *p*-DTS(FBTTh_2_)_2_ and PC_71_BM blends. In the presence of DIO, which is less volatile than the parent solvent (CB), evaporation of the additive leads to the second path along a direction out-of-plane due to further evaporation of DIO until the system runs out of the parent solvent completely. Complete evaporation of the additive brings DIO- *p*-DTS(FBTTh_2_)_2_:PC_71_BM plane to the final blend of *p*-DTS(FBTTh_2_)_2_:PC_71_BM. These thermodynamic calculations highlight that although the nature of the final stage is the same, independent of the presence or absence of solvent additives, the kinetic processes (such as evaporation rates, mobility of *p*-DTS(FBTTh_2_)_2_ and PC_71_BM) are important to differentiate between the morphological differences. For example, Table 3 reveals that both the CB and DIO are non-selective solvents for the *p*-DTS(FBTTh_2_)_2_ and PC_71_BM (i.e. χ_DTS,CB_ = χ_PCBM,CB,_ χ_DTS,DIO_ = χ_PCBM,DIO_) suggesting that the CB as well as DIO will stabilize interfaces between *p*-DTS(FBTTh_2_)_2_ and PC_71_BM domains due to entropic effects. However, χ_DTS,CB_ is less than χ_DTS,DIO_ suggesting that DIO results in better entropic stabilization of interfaces. The latter is in qualitative agreement with the X-ray diffraction analysis, which shows that DIO increases population of smaller crystalline domain size of *p*-DTS(FBTTh_2_)_2_ aggregates in *p*-DTS(FBTTh_2_)_2_:PC_71_BM films. In this study, we have developed and proposed a strategy by combining experimental and theoretical approach. This approach allows us to demonstrate the correlation between the morphology and device performances as well as the origin of different morphologies. Such information may aid in improving the morphology and to enhance the device performance for OPV devices.

## Conclusions

We report that the thermodynamically stable *p-*DTS(FBTTh_2_)_2_:PC_71_BM BHJs obtained by thermal annealing are different in lateral and depth phase morphology and crystallinity from BHJs obtained with a DIO additive. Through an energy-level analysis on the absorption and emission spectra of the films, the different functions of thermal or DIO treatment to the π−π stacking in *p-*DTS(FBTTh_2_) aggregates and the interaction at *p-*DTS(FBTTh_2_)_2_-PC_71_BM interface have also been revealed. The three-layer vertical phase morphology was observed for the films after thermal annealing. By contrast, DIO additive processing generates more evolved film morphology, which is closer to the equilibrium state. *n*SLD chemical/structural profiles obtained from NR data show more densely packed structures, which are consistent with the AFM images. Formation of well-mixed ED and EA regions at the surface of the film facilitates the efficient charge transfer to the device. According to X-ray diffraction data, the DIO additive morphology exhibits a high density of small donor nanocrystallites of ~24 nm, whereas thermal annealing generates smaller amount of much larger crystallites (~134 nm). DIO promotes the formation of a large density of *p*-DTS(FBTTh_2_)_2_ small nanocrystals arranged in an elongated network throughout the thickness of the active layer, and it results in the enhancements of solar cell performance due to the formation of nanocrystallites with the domain size comparable to the maximum exciton diffusion length. This is favorable for device efficiencies, since they stimulate efficient exciton diffusion (less probable to recombine) to an ED/EA interface and provide a larger ED/EA interfacial area for exciton dissociation. Thermodynamic analysis based on the Flory-Huggins theory for the experimentally studied systems reveals that the CB as well as DIO act as non-selective solvents for the *p-*DTS(FBTTh_2_)_2_:PC_71_BM blends (i.e., χ_DTS,CB_ = χ_PCBM,CB,_ χ_DTS,DIO_ = χ_PCBM,DIO_). However, DIO has an increased tendency to induce phase segregation resulting from the fact that χ_DTS,CB_ < χ_DTS,DIO._ Furthermore, the inverse temperature dependence of the χ parameters allows us to infer the effects of temperature on the phase segregation, which is in qualitative agreement with the observed morphological changes. Our findings demonstrate the role and impact of DIO and thermal treatment on the morphology of small molecule BHJ and take us a step closer to fully controlling the performance of photovoltaic devices.

## Methods

### Devices fabrication

*p*-DTS(FBTTh_2_)_2_ and PC_71_BM were purchased from “1-Material”. The blends of *p-*DTS(FBTTh_2_)_2_ (21 mg) and PC_71_BM (14 mg) were dissolved in chlorobenzene (1 ml) with and without 0.25 Vol.% diiodooctane. Solutions were heated at 60 °C for several hrs and 90 °C for 15 min just before spin casting. Devices were fabricated as follows: ITO substrates were first cleaned by detergent and subsequently sonicated in DI water, acetone, and isopropyl alcohol (IPA), followed by backing at 100 °C for an hr. The cleaned ITO substrates were then treated with UV Ozone for 20 min, and PEDOT:PSS solution was spun-cast on them and baked for 1 hour at 135 °C in air. The blend solutions with and without DIO were spun-cast on the PEDOT:PSS-coated substrates at a spinning speed of 2000 rpm for 45 s. Films were allowed to dry for 20 min under inert atmosphere and annealed at 80 °C for 10 min. Devices without DIO were annealed at either 80° or 100 °C for 10 min. Finally, Ca and Al electrodes were deposited on top of the active layers through a shadow mask by the thermal evaporation. The electrode area of the cells was 22.6 mm^2^. The J-V characteristics of the prepared devices were measured by a Keithley 4200 semiconductor parameter analyzer under the AM 1.5 conditions (100 mW/cm^2^).

The morphological characterizations were done with blends of *p-*DTS(FBTTh_2_)_2_:PC_71_BM BHJ spun cast on sapphire (Al_2_O_3_) or quartz (SiO_2_) substrates. To obtain the neutron scattering density (nSLD) of pure *p-*DTS(FBTTh_2_)_2_, a solution of *p-*DTS(FBTTh_2_)_2_ (12 mg/ml) in chlorobenzene was prepared and spun-cast onto a silicon wafer.

## Thin film characterizations

### Neutron reflectomety

The neutron reflectometry experiments were performed at the Magnetism Reflectometer (BL 4A) and the Liquid Reflectometer (BL 4B) at the Spallation Neutron Source at Oak Ridge National Laboratory. The data were recorded on position sensitive detectors and the reflected and scattered intensity signals were normalized to the intensity spectrum of the incident beam. The data are presented in two-dimensional maps as a function of *p*_i_ and *p*_f_ where *p*_i_ = 2*π* sin*α*_i_/*λ* and *p*_f_ = 2*π* sin*α*_j_/*λ* are the perpendicular components of the neutron wave vectors. The specular reflectivities are extracted from these two-dimensional intensity maps as a function of incident momentum transfer normal to the surface, Q_z_ = p_i_ + p_j_ = 4π sinα_i_/λ. The experiential data is used to extract neutron scattering length density (*n*SLD) values.

### X-ray Diffraction and X-ray reflectomety

Measurements were carried out on a high-resolution PANalytical X’Pert Pro MPD diffractometer with a Cu Kα source (wavelength 1.5405 Å). The X-ray diffraction measurements were performed at 2.5–35 Å angular range with 0.04° step size and 0.5° scan speed. Standard single crystal silicon single crystal sample was used measured to calibrate the instrument.

### UV-Visible absorption and PL measurements

UV-Vis absorption spectra (Abs) were recorded using a Cary 5000 spectrometer from the film obtained by solution spin-casting and subsequent thermal processing. Photoluminescence (PL) was measured using a FluoroLog 3 T fluorescence spectrometer, the excitation monochromator was set at 320 nm and 580 nm.

### AFM

Atomic Force microscopy was recorded using Bruker Dimension Icon operating in a tapping mode.

## Additional Information

**How to cite this article**: Herath, N. *et al.* Peculiarity of Two Thermodynamically-Stable Morphologies and Their Impact on the Efficiency of Small Molecule Bulk Heterojunction Solar Cells. *Sci. Rep.* **5**, 13407; doi: 10.1038/srep13407 (2015).

## Supplementary Material

Supplementary Information

## Figures and Tables

**Table 1:** Peak area and the crystallite sizes for *p-*DTS(FBTTh_2_)_2_ (001) reflection.

**Sample**	Normalized PeakArea	**Crystallite size(nm)**
As cast	2205	22.8
Annealed at 80 °C	2358	41.2
Annealed at 100 °C	3189	134.7
With 0.25 V% DIO	6687	24.6
With 0.25 V% DIO ann. 80 °C	7386	26.4

**Table 2:** Electrical Parameters of *p-*DTS(FBTTh_2_)_2_:PC_70_BM solar cells at different processing conditions.

**Device**	J_SCavg_(mA/cm^2^)	**V_OCavg_(V)**	**FF_avg_(%)**	**PCE_avg_ (%)**
As cast	6.66	0.81	37.84	2.04 ± 0.29
Annealed at 80 °C	7.62	0.81	51.40	3.18 ± 0.30
Annealed at 100 °C	7.85	0.81	54.60	3.48 ± 0.32
With 0.25 V% DIO	11.74	0.78	54.24	4.95 ± 0.07
With 0.25 V% DIO ann. 80 °C	12.01	0.76	58.15	5.27 ± 0.48

Values are obtained from averaging parameters from ten devices.

**Table 3:** Estimated χ_ij_ for different pairs along with their values (in parenthesis) at T = 298 K, relevant for this study.

**Parameter**	***i* = p-DTS,*j* = PC**_**71**_**BM**	***i* = DTS, *j* = CB**	***i* = DTS, *j* = DIO**	***i* = PC_71_BM,*j* = CB**	***i* = PC_71_BM,*j* = DIO**	***i* = CB, *j* = DIO**
χ_ij_	0.34 + 13.19/T (0.384)	0.34 + 90.64/T (0.644)	0.34 + 436.37/T (1.804)	0.34 + 108.44/T (0.703)	0.34 + 431.65/T (1.788)	0.34 + 29.50/T (0.439)
