# Supplementary material for: Peculiarity of Two Thermodynamically-Stable Morphologies and Their Impact on the Efficiency of Small Molecule Bulk Heterojunction Solar Cells
Source: Sci Rep. 2015 Aug 28;5:13407. doi: 10.1038/srep13407 (PMC4642552; doi:10.1038/srep13407)
Supplement: Supplementary Information [file srep13407-s1.pdf]

Supplementary materials

Peculiarity of Two Thermodynamically-Stable Morphologies and Their Impact on the Efficiency Small Molecule Bulk Heterojunction Solar Cells

Nuradhika Herath[1], Sanjib Das[2], Jong K. Keum[3], Jiahua Zhu[3], Rajeev Kumar[3,5], Ilia N. Ivanov[3], Bobby G. Sumpter[3,5], James F. Browning[4], Kai Xiao[3], Gong Gu[2], Pooran Joshi[6] and Valeria Lauter[1*]*

[1]Quantum Condensed Matter Division, Oak Ridge National Laboratory, Oak Ridge, TN 37831.USA
[2]Department of Electrical Engineering and Computer Science, University of Tennessee, Knoxville, TN 37916, USA
[3]Center for Nanophase Materials Sciences, Oak Ridge National Laboratory, Oak Ridge, TN 37831,USA
[4]Chemical and Engineering Material Division, Oak Ridge National Laboratory, Oak Ridge, TN 37831, USA
[5]Computer Science and Mathematics Division, Oak Ridge National Laboratory, Oak Ridge, TN, 37831, USA
[6]Materials Science and Technology Division, Oak Ridge National Laboratory, Oak Ridge, TN 37831, USA.

Corresponding Authors: herathnn@ornl.gov, lauterv@ornl.gov

Neutron reflectometry on SiO_2/Al_2O_3 substrates and on PEDOT:PSS layer

Initially, we carried out NR measurements of *p*-DTS(FBTTh2)2:PC_{71}BM BHJ films on two different substrates (SiO_2 and Al_2O_3) and compared *n*SLD profiles extracted from the data. As illustrated by Figure 1, the *n*SLD profiles show presence of three-layer morphology. For both samples, we observed presence high concentration of *p*-DTS($FBTTh_2$)$_2$ at two interfacial layers and the sandwich layer containing 60:40 mixture of DTS($FBTTh_2$)$_2$:PC_{71}BM. Hence, the vertical morphologies of the *p*-DTS(FBTTh2)2:PC_{71}BM BHJ films do not depend on the substrate.

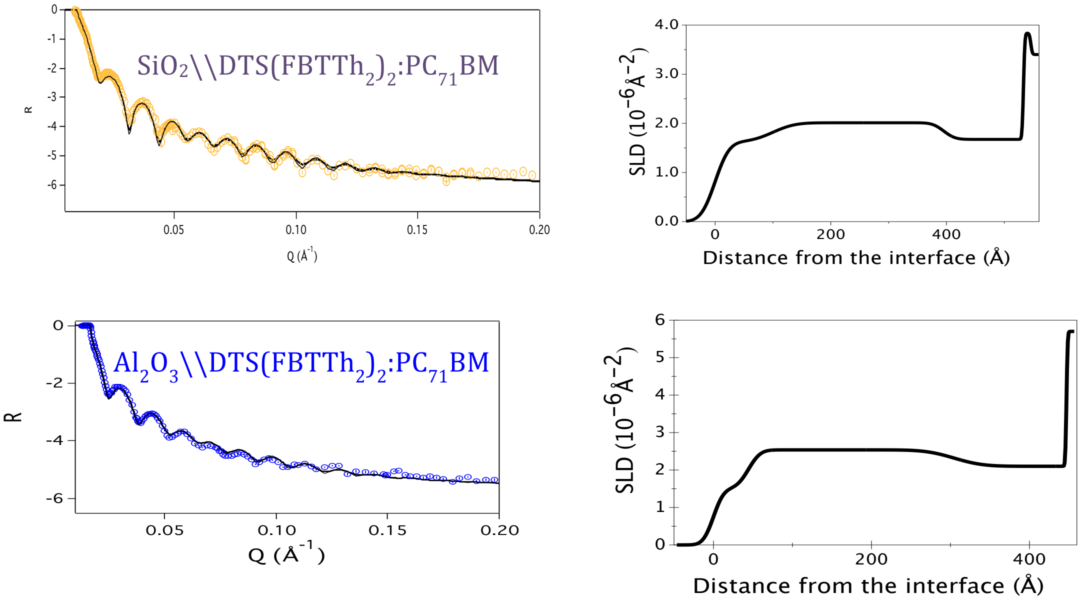

Figure S1: (a) Neutron reflectivity data of SiO_2\\DTS($FBTTh_2$)$_2$:PC_{71}BM BHJ device as cast film fitted using a single layer model (b) SLD profile extracted from the fitting (a), (c) Neutron reflectivity data of Al_2O_3\\ *p*-DTS($FBTTh_2$)$_2$:PC_{71}BM BHJ device as cast film fitted using a single layer model (d SLD profile extracted from the fitting (c)

The same vertical morphology was observed for the sample containing PEDOT:PSS layer, Al_2O_3\\PEDOT:PSS\DTS($FBTTh_2$)$_2$:PC_{71}BM (Figure 2). We observed three layer morphology of *p*-DTS($FBTTh_2$)$_2$:PC_{71}BM despite the presence of PEDOT:PSS layer. Therefore, PEDOT:PSS layer does not change the vertical morphology of the system and our results accurately reflect the vertical phase morphology of the OPV devices

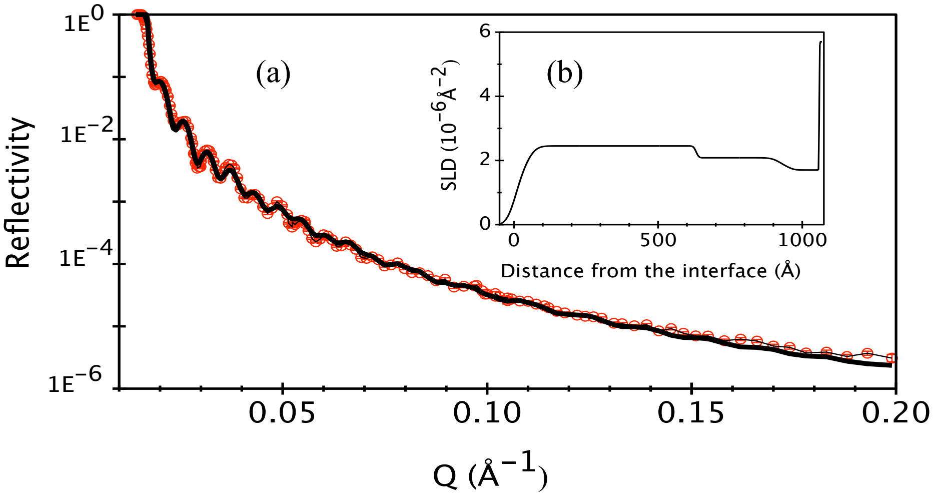

Figure S2: (a) Neutron reflectivity data of Al_2O_3\\PEDOT:PSS\DTS(FBTTh$_2$)$_2$:PC$_{71}$BM BHJ device as cast film fitted using a single layer model (b) SLD profile extracted from the fitting (a)

Fitting Neutron reflectivity data using Parratt recursion formalism:

The neutron reflectivity data were fitted into several models and the model that gives the best fit was used to obtain *n*SLD profiles. A single layer model consisting a uniform layer of the active layer was used to fit the refelctometry curves (Figure S1 (a)). As illustrated by Figure S1 (a), the single layer model does not provide the best fit for the data, indicating layer separation during the spin coating. Hence, a complex model was required to obtain a better fit for data. Consequently, two and three layer models were used to fit the data. The best fit for NR data was obtained when three-layer model was used (Figure S1 (c)). Adding another layer (four-layer) did not show further improvement for the fit. Therefore, three-layer model was considered to get *n*SLD profiles for NR data.

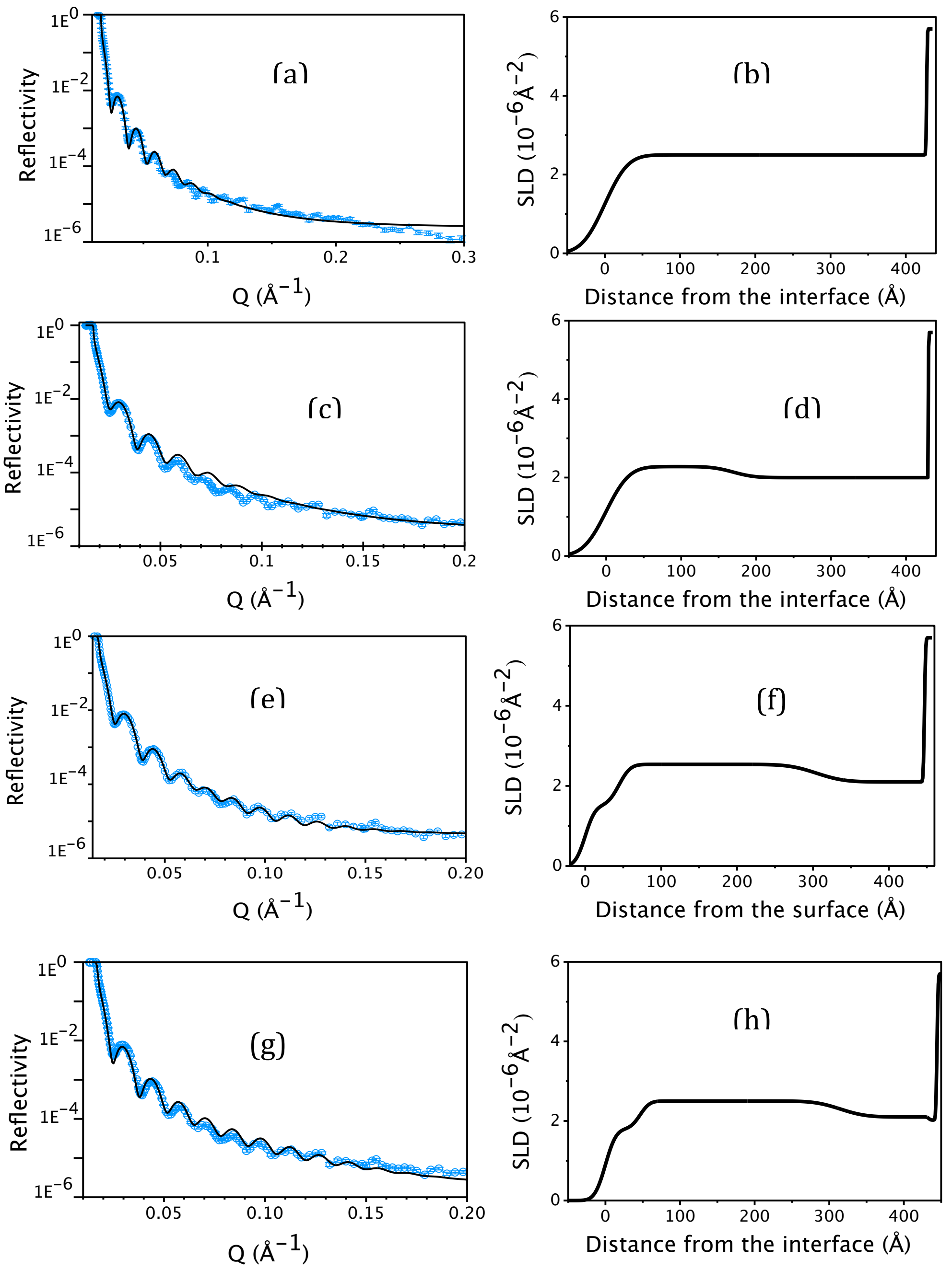

Figure S3: (a) Neutron reflectivity data of Al_2O_3*p*-DTS(FBTTh$_2$)$_2$:PC$_{71}$BM BHJ device as cast film fitted using a single layer model (b) SLD profile extracted from the fitting (a), (c) Neutron reflectivity data of Al_2O_3\\ *p*-DTS(FBTTh$_2$)$_2$:PC$_{71}$BM BHJ device as cast film fitted using a two- layer model (d) SLD profile extracted from the fitting (c), (e) Neutron reflectivity data of Al_2O_3\\ *p*-DTS(FBTTh$_2$)$_2$:PC$_{71}$BM BHJ device as cast film fitted using a three layer model (f) SLD profile extracted from the fitting (e), (g) Neutron reflectivity data of Al_2O_3\\ *p*-DTS(FBTTh$_2$)$_2$:PC$_{71}$BM BHJ device as cast film fitted using a three layer model (h) SLD profile extracted from the fitting (g)

(a)

(b)

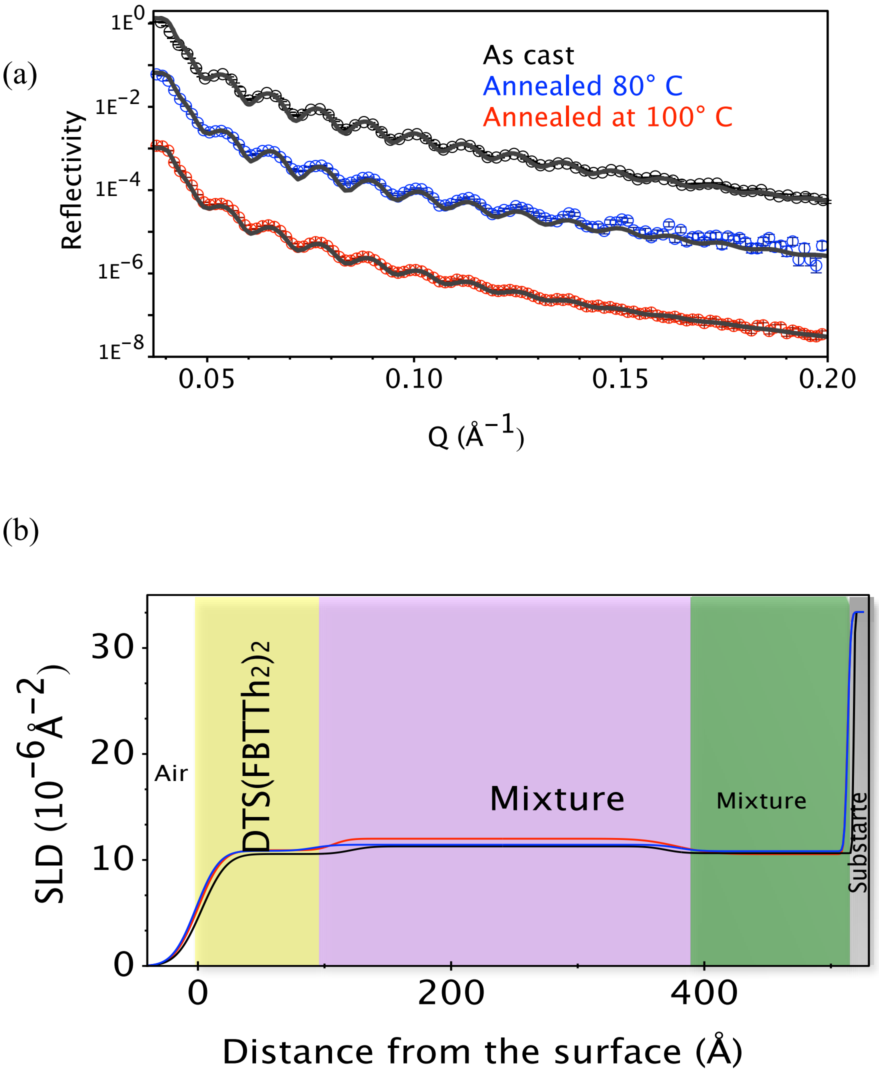

Figure S4: (a) X-ray reflectivity (XRR) profiles of Al_2O_3*p*-DTS(FBTTh$_2$)$_2$:PC$_{71}$BM BHJ test structures as prepared and annealed at 80° C, 100° C. Experimental data are shown as symbols and the fits as lines. (b) Scattering length density (SLD) profiles as a function of distance from the interface from fitting the experimental XRR data of (a).

Theoretical calculation phase segregation using Flory-Huggins[1] theory: For *p*-DTS(FBTTh$_2$)$_2$:PC$_{71}$BM system, we have estimated the Flory's χ_{ij} parameters by the simple relation[2], $\chi_{ij} = 0.34 + (v_i v_j)^{1/2}(\delta_i - \delta_j)^2/RT$, where R and T are the gas constant and temperature (in Kelvin), respectively. v_i and δ_i are the molar volume and the solubility parameter of component *i*, respectively, which are taken either from literature[3,4] or estimated using the group contribution method of Hoy[2] (cf. Table S1)

Table S1. Structural parameters for different molecules studied in this work

Parameter	*p*-DTS(FBTTh$_2$)$_2$[a] ($C_{64}H_{72}F_2N_4S_8Si$)	PC$_{71}$BM[b] ($C_{82}H_{14}O_2$)	DIO[c] ($C_8H_{16}I_2$)	CB[c] (C_6H_5Cl)
δ_i [(J/cm^3)$^{1/2}$/mole]	20.96	21.34	18.12	19.43
v_i [cm^3/mole]	1006.7	600.0	198.9	101.8

[a]: using the method of Hoy as described in Ref. 2; [b]: Ref. 3; [c]: Ref. 4.

The quaternary phase diagram calculations are done by direct minimization of the Flory-Huggins expression for the free energy with respect to the four unknowns: the volume fraction of any three components in one phase and volume fraction of one of the coexisting phases. Other unknowns are readily calculated using the lever rule and incompressibility condition[1]. For these calculations, we have treated *p*-DTS(FBTTh$_2$)$_2$ and PC$_{71}$BM as oligomers with N=10 and N=6, respectively, as the degree of polymerization to account for disparity in their molar volume with respect to the solvent chlorobenzene (cf. Table S1). This particular choice of the parameters doesn't affect the phase diagram significantly, that is, the overall shape of the diagram stays the same.

References

1 Flory, P. J. *Principles of Polymer Chemistry*. (Cornell University Press, 1953).

2 van Krevelen, D. W. *Properties of Polymers*. (Elsevier, 1990).

3 Yaws, C. L. *Thermophysical Properties of Chemicals and Hydrocarbons*. (Elsevier, 2008).

4 Walker, B. T., A.; Duong, D. T.; Dang, X. D.; Kim, C.; Granstrom, J.; Nguyen, T. Q. A systematic approach to solvent selection based on cohesive energy densities in a molecular bulk heterojunction system. *Adv. Ener. Mater.* **1**, 221-230 (2011).
